# A genetic-based pairwise trip planner recommender system

**DOI:** 10.1186/s40537-021-00470-6

**Published:** 2021-05-30

**Authors:** Nunung Nurul Qomariyah, Dimitar Kazakov

**Affiliations:** 1 Computer Science Department, Faculty of Computing and Media, Bina Nusantara University, Jakarta, Indonesia 11480; 2 Computer Science Department, University of York, York, UK

**Keywords:** Recommender system, Pairwise preferences, Genetic algorithm, Preference learning

## Abstract

The massive growth of internet users nowadays can be a big opportunity for the businesses to promote their services. This opportunity is not only for e-commerce, but also for other e-services, such as e-tourism. In this paper, we propose an approach of personalized recommender system with pairwise preference elicitation for the e-tourism domain area. We used a combination of Genetic Agorithm with pairwise user preference elicitation approach. The advantages of pairwise preference elicitation method, as opposed to the pointwise method, have been shown in many studies, including to reduce incosistency and confusion of a rating number. We also performed a user evaluation study by inviting 24 participants to examine the proposed system and publish the POIs dataset which contains 201 attractions used in this study.

## Introduction

The rapid growth of internet users nowadays has impacted many countries in the world. This growth also occurred in Southeast Asia. In a most recent published annual report by Google, Temasek and Bain & Company on Southeast Asia’s (SEA) digital economy, titled *e-Conomy SEA 2020* [1], it has been revealed that there is a massive increasing amount of internet users with 40 millions of new users in the year 2020 alone. The total of active internet users in SEA has reached 400 million in 2020. The outbreak of COVID-19 was considered to be the major reason for the digital market acceleration. The new users tried to use digital services to fullfil their need during the pandemic and the countries’ lockdown policy. This new phenomenon can have a good impact on the country’s economy as this new trend seems to be sticky. Of all those new digital internet users, it has been reported that the 94% of them intend to continue with the current service even after the pandemic situation is over. This is a good opportunity for businesses to keep improving their digital services.

A smart way to improve digital businesses has been proven by big web services companies like Youtube, Amazon, Netflix, Instagram and Facebook through the implementation of the recommender systems. They are a technology to suggest the most suitable contents to their users and match with the user preferences. During the last couple of decades, it was no doubt that the implementation of this technology was able to make them stand out significantly from their competitors. In addition to that, recommender systems are not only beneficial for the businesses, but also for the users [2]. For the users, they can minimize the cost of choosing the most suitable product in the context of internet shopping [3]. The example of costs that can be minimized is the cost of buying the wrong and unnecessary services from the wrong providers and costs of browsing. Another benefit for the users, as mentioned by Pathak et al. [4], is that the recommender systems can improve the decision making process and quality.

Currently, there are many various techniques used to build the recommender systems such as collaborative filtering, content-based filering and hybrid filtering [5]. The Collaborative Filtering (CF) technique is the most well-known and most commonly implemented in industries. It works by recommending the items based on the other users with similar taste. The second most commonly used is Content-Based (CB) filtering. This technique works by recommending similar items based on the particular user’s information without taking into consideration the other users. There is also a hybrid filtering that combines more than one technique of recommender system algorithms.

Most of the common approaches use *single point feedback* as the input to the system such as rating score with a linear scale of 1 to 5 (1 for the worst and 5 for the best). Based on these ratings, the system can calculate the recommendation score for each other item. although has been very well implemented, this single point feedback approach still has some drawbacks as mentioned in [6], such as the ratings are quite personal. Even though two users have similar preferences, it is very likely they will give different ratings to the same items. The other drawback is inconsistency. Users easily forget what ratings did they give for the items with similar properties in the past, so whenever they are asked to give a rating to the similar items, they can give a different score. A user may also feel difficult to give a slightly lower preference to some items because the rating scale does not normally have a half score. Therefore, there is another approach, called pairwise elicitation, which has been introduced by the researchers in this domain which shows pair choices to the users. The problem of eliciting preference by using the pairwise method itself still become an interest for the researchers, such as in [6–13]. By using this approach, a user will be shown with a series of pair options and a preference can be expressed by selecting only one of the most preferred item between the two items (a pair) at a time. This technique can reduce the confusion of a rating number. In this paper, we want to focus on the use of the pairwise elicitation method to learn about user preferences. Some studies have also shown the combination of the pairwise elicitation with the use of CF as well as the CB filtering method in a recommender system, such as the one introduced by Liu et al. [7]. Our study will propose a different approach, i.e. using a genetic algorithm to optimize the searching strategy combined with pairwise preferences as the elicitation method.

Recommender systems are very popular nowadays due to the benefit being offered as explained earlier. In an e-commerce context, they can reduce the cost of searching and finding suitable items and direct the customers to buy products they mostly liked. Similar to that, in the tourism domain, the travellers often face the problem of spending too much time browsing the possible destinations before visiting a new place which can waste their time and energy. The searching action can be more complicated in the case of the limited budget and the trip duration which can be spent by the travellers. They need to choose the destinations wisely so that they still can spend the money and time in an effective way. One possible solution is by using Google Maps and choose the destination manually. We can see there is a gap between the existing solution and the research to address this problem. Often with the same difficulties as faced by the e-commerce users, the travel application users also feel overwhelmed with many choices available. The pairwise choices can simplify the options and generate a list of recommendation even before they provide ratings to the items. This is one of the advantages of our proposed approach.

In 2020, we proposed a system design of an e-tourism mobile application as a solution to promote tourism in Indonesia which has been worsened due to the COVID-19 pandemic situation, called SONIA (*pariwiSata ONline IndonesiA* or Indonesia Online Tourism) [14]. In this paper, we will discuss in more detail the recommender system module of the system. In summary, the contributions of this study are as follows: (1) publish a dataset of tourist attractions in Jakarta, Indonesia to be used for further study, (2) propose an approach of a trip recommender system based on a genetic algorithm with pairwise options, and (3) conduct a user evaluation study on the implemented pairwise trip recommender system with real-life data.

## Related work

Genetic Algorithm (GA) has been around for decades, but the use of this optimization algorithm in recommender system is still limited. GA has been used in many domains with optimization and search problems. In general, a recommendation problem can also be considered as a searching problem for the best items. All the recommender system algorithms aim to give as least error as possible. GA is believed to be an effective algorithm that can provide a near-optimal solution in a reasonable time. Henceforth, there is no reason why GA is not suitable for solving a recommendation problem.

In the Recommender System (RS) domain, GA has been utilized for clustering, such as the study performed by Kim and Ahn in 2008 [15], Zhang and Chan in 2006 [16], and Mohammadpour et al. in 2019 [17]. The researchers in this area also used GA to increase the accuracy of recommendation proposal generated by classic RS algorithms, such as collaborative filtering and content-based filtering. GA works in improving the population of recommendation solutions in each iteration. A study by Kilani et al. [18] proposed the GA-based matrix factorization hybrid approach of RS. They use the approach to predict items for the active user. This is an improvement work of a study by Navgaran et al. [19]. They show that their approach can perform faster than the previous work with better recall and precision values in some datasets. Recently, Alhijawi and Kilani in 2020 [20] proposed a novel GA-based collaborative filtering that aims to select the best items which meet the active user’s preferences based on multi-filtering criteria. Another recent study by Gasmi et al. in 2021 [21] also proposed a user-based collaborative filtering combined with the GA based meta-heuristic. A study by Xiao [22] proposed a combination of item-based collaborative filtering with GA which is called itemCFGA. This study also proposed a novel similarity function that uses the average rating of each user. For a hybrid RS model, GA is used in [23] and  [24]. Another related work in this area has been introduced in [25] and [26].

## Jakarta tourist destination dataset

As our first contribution, we share the dataset we have collected and used in this study. The dataset was built by scraping information from many sources in the period between May–June 2020. The data has been checked again for the updated one by the time of submitting this manuscript on April 2021. The dataset focuses on the famous tourist destinations in Jakarta, Indonesia. It was the time when the COVID-19 outbreak was still affecting the country. Due to the lockdown policy, the data of some entrance fees, opening and closing hours, may be different from the regular condition. We will keep updating the data once the condition is already back to normal. The dataset consists of 201 POI entries, with 10 columns. Each of the dataset columns is described in Table 1. We also published the distance matrix together with duration matrix data which were collected by using Google Maps API. The sample of the distance matrix database is shown in Table 2 and the duration matrix is shown in Table 3.

**Table 1:** Places dataset description

Column Name	Descriptions	Example Value
Address	Address of the places	Gambir, Central Jakarta City, Jakarta, Indonesia
Geometry	Geographic coordinate	{’location’: {’lat’: -6.1753, ’lng’: 106.8271}
		’viewport’: {’northeast’: {’lat’: -6.1672, ’lng’: 106.8341}
		’southwest’: {’lat’: -6.1845, ’lng’: 106.8196}}}
Name	Places name	National Monument
Place_id	Google maps places ID	ChIJLbFk59L1aS4RyLzp4OHWKj0
Rating	User ratings (1-5)	4.6
Types	Google maps place types	[’tourist_attraction’, ’point_of_interest’, ’establishment’]
Url	Google maps URL	https://maps.google.com/?cid=4407571450964851912
PictureSource	Image URL	https://upload.wikimedia.org/wikipedia/commons/b/b1/Merdeka_Square_Monas_02.jpg
TicketPrice	Entrance fee (in IDR)	20000
	For one regular adult	
VisitDuration	Most people time spent	0.5
	(in hours)	
Tags	Manually labelled	[’kids-friendly’, ’history’]
	Place category

**Table 2:** Sample of Distance Matrix (in metres)

Origin	Travel Mangroove	Apartemen	Waduk Setia	Aqua Fun
	Forests	Taman Melati	Budi Timur	
Travel Mangroove Forests	0	54682	43132	35001
Apartemen Taman Melati	49698	0	22246	46348
Waduk Setia Budi Timur	36291	24067	0	22424
Aqua Fun	31918	43536	22062	0

**Table 3:** Sample of duration matrix (in seconds)

Origin	Travel Mangroove	Apartemen	Waduk Setia	Aqua Fun
	Forests	Taman Melati	Budi Timur	
Travel Mangroove Forests	0	5084	4035	3585
Apartemen Taman Melati	4604	0	2287	2741
Waduk Setia Budi Timur	3799	2325	0	1562
Aqua Fun	3938	3116	1988	0

## The proposed system

### System architecture

**Fig. 1:**
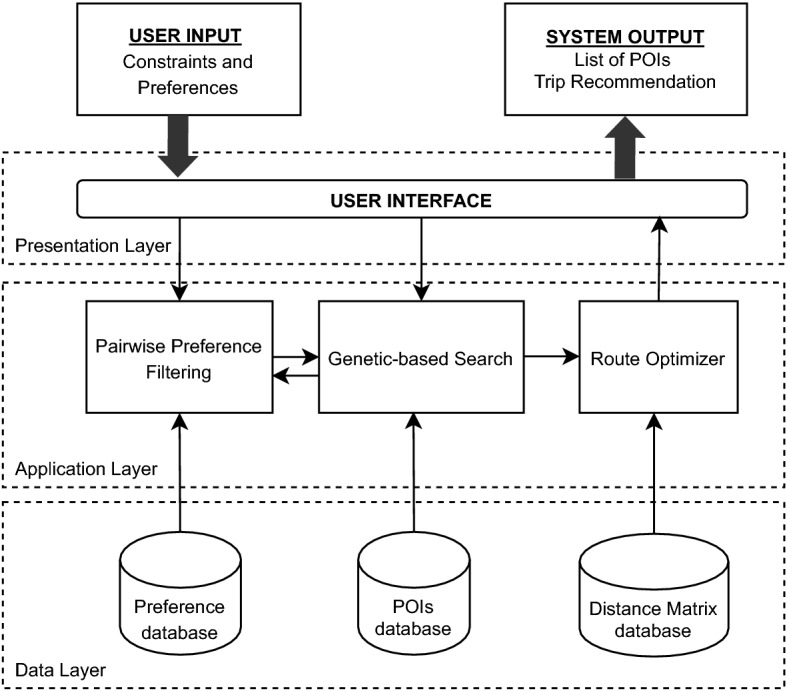
Proposed recommender system architecture

The main architecture of the proposed system is shown in Figure 1. We propose the use of a three layers design architecture, which contains the presentation layer (frontend), application layer (backend) and data layer (database). In the presentation layer, the user will communicate through a user interface, where constraint and preference will be the inputs and list of Point of Interests (POIs) recommendation will be the output of the system. In the application layer, there are three main components, namely pairwise preference filtering, genetic-based search, and route optimizer. In the data layer, there are three databases connected to the system, they are to store preference, POIs and distance matrix.

### System flows

**Fig. 2:**
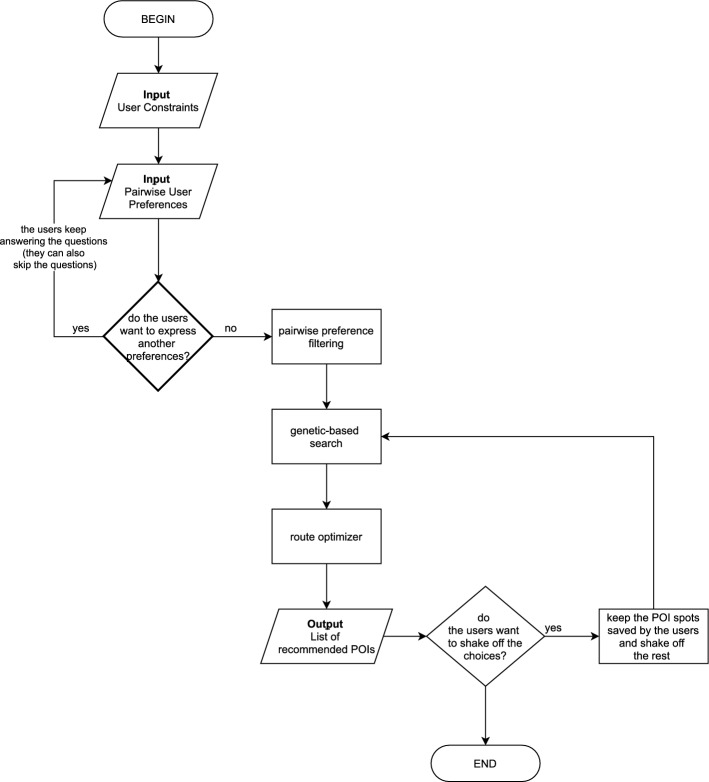
Proposed recommender system flowchart

In this section, we will explain the general flow of the system. A more detailed explanation of each process will be described in the next section. The system flowchart of the proposed recommender systems is shown in Fig. 2.

**Fig. 3:**
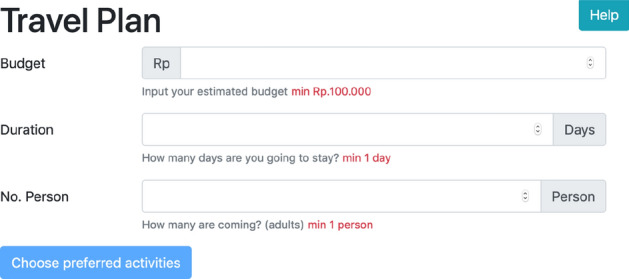
UI Design for user constraints input

**Fig. 4:**
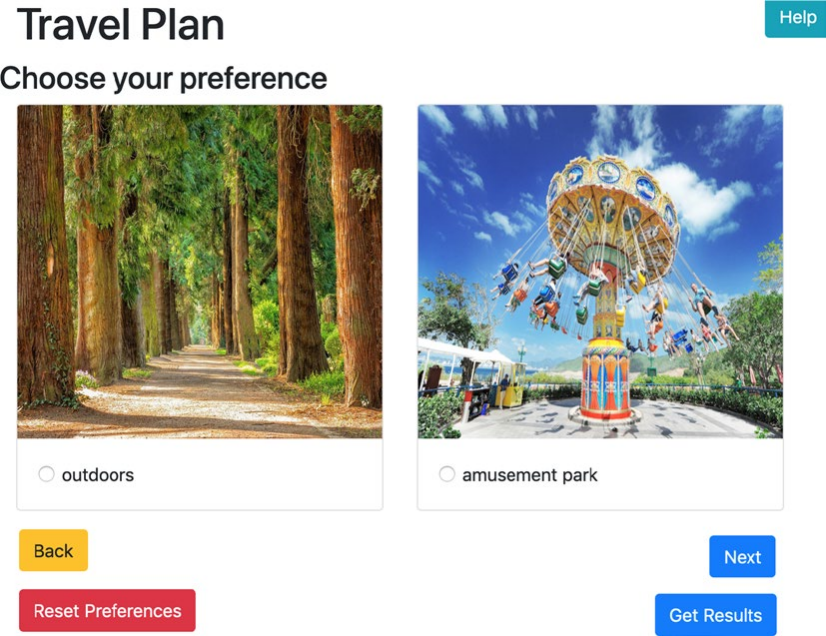
UI design for user pairwise preferences input

The system has two different inputs, are (1) user constraints, and (2) pairwise user preferences. The system starts with an input form for expressing the user scope of travel budget, travel duration, and the number of adults joining the group. This form is mandatory, so when a user is not willing to fill in these three mentioned constraints, the system will not proceed to the next page. The UI design is shown in Fig. 3. On the next screen, the user will be asked to express their preference of destinations by choosing one best of the two choices shown. The users will have the options to skip the question and move to the next question, or just skip and go straight to the result page. The choices shown to the users are randomly selected by the algorithm. For this input, the UI design is shown in Fig. 4.

If the users want to stop inputting the preferences, they have the option to proceed to the next process which is the pairwise preference filtering process. The system will filter the eligible POIs and pool them together for the next process. After this step has been done, the system will pass the pool of eligible POIs to the next process, genetic-based search. Here the optimized search algorithm works to find the best combination of POIs based on the constraints given by the users. The next step is, pass the result to the route optimizer, which will find the best route for the users.

The system will show the list of recommended POIs, arranged in the best way for each day of travel. The process does not end here. The users still have the freedom to choose whether they already satisfied with the recommendation list or not. They have the option to keep some of the recommended POIs and repeat the search with the rest constraints. This last step can be done multiple times.

### Presentation layer

**Fig. 5:**
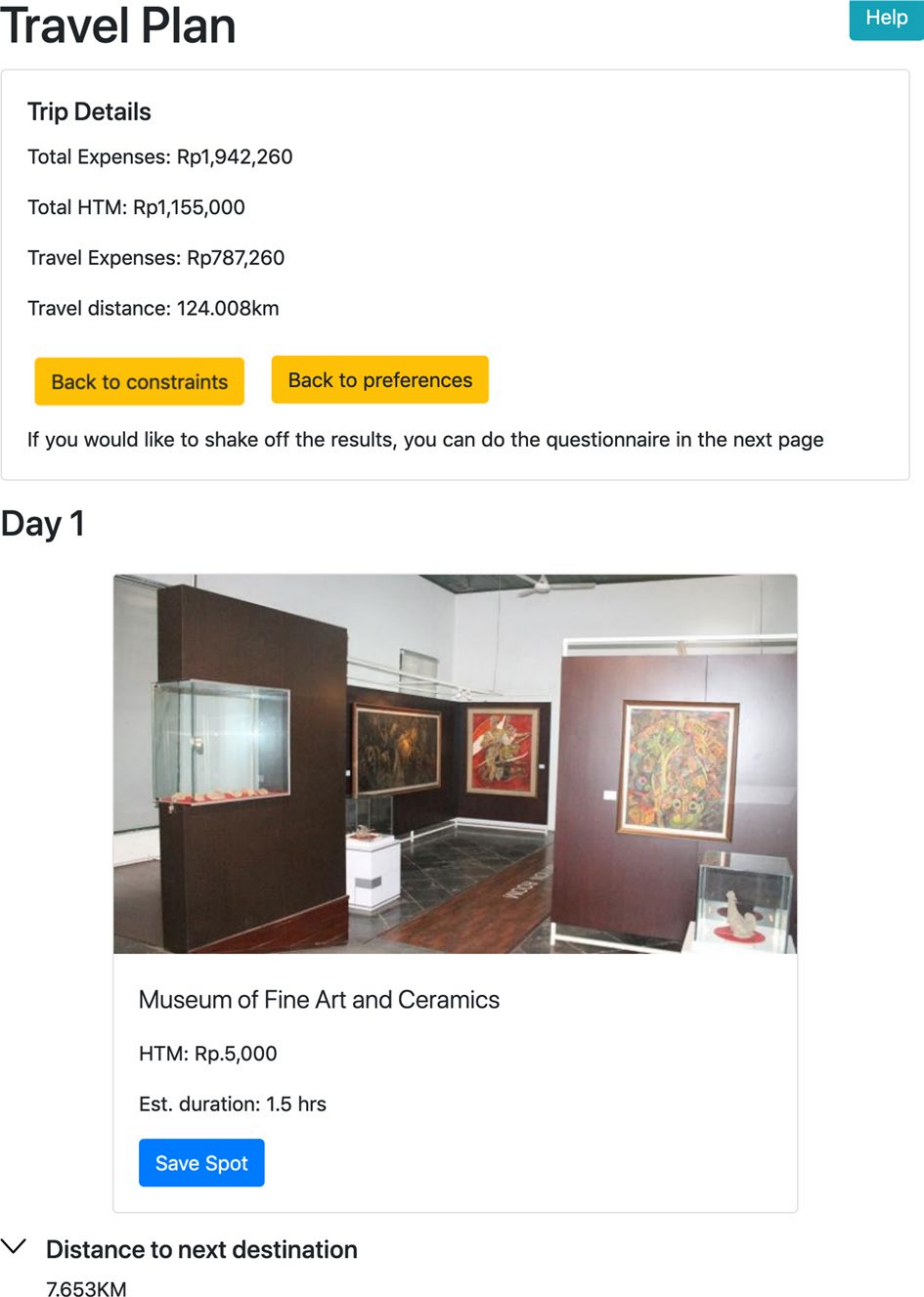
UI design for list of trip recommendation

**Fig. 6:**
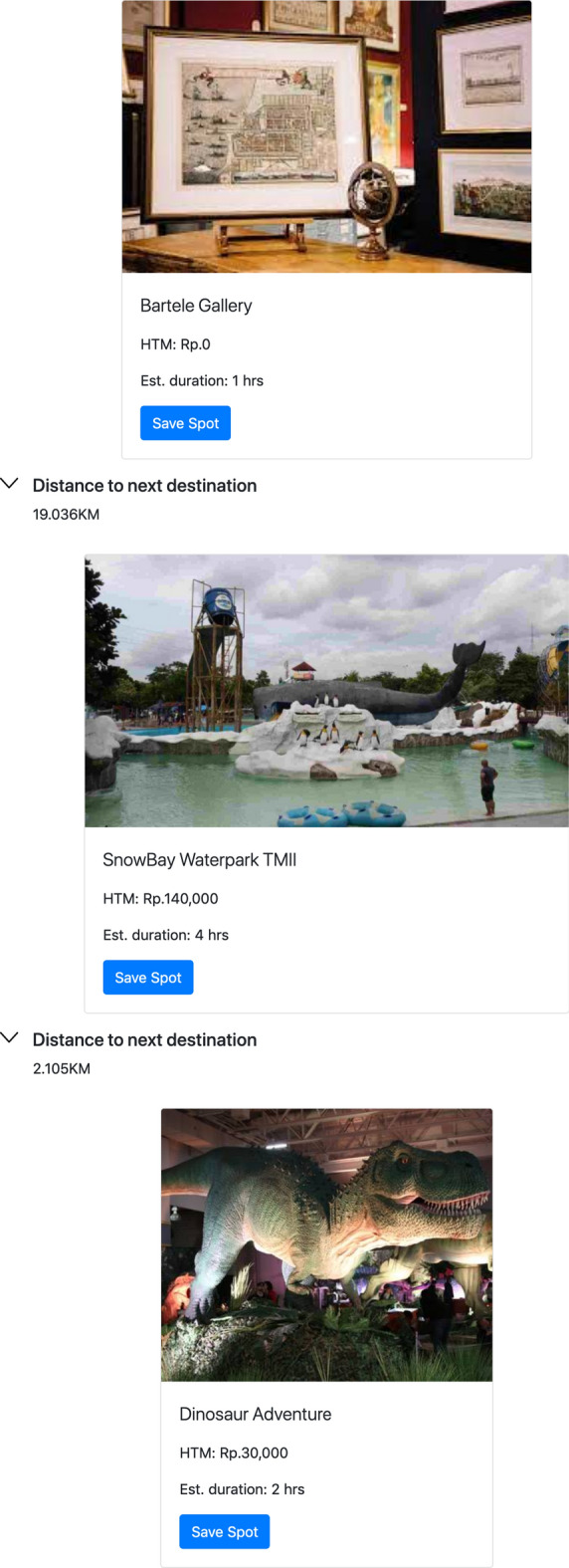
UI design for list of trip recommendation (cont.)

In this layer, we propose a simple design to communicate with the users. We are not designing a recommender system as a standalone system, but as a module of a big e-tourism system. The basic User Interface (UI) design can be integrated into the main application easily, following the main design template. There are three main pages needed in this proposed recommender system, i.e. UI for user constraints, UI for user preferences, and UI for the recommendation result. The user interface design is shown in Figs. 3, 4, 5 and 6.

Figure 3 shows the first part of the user input. This is where the users can decide their budget, duration and number of persons in a travel group. Based on these constraints, the system will calculate the most suited travel destinations to visit. The minimum budget is IDR 100,000, the minimum duration is 1 day, and the minimum number of person in a group is 1 adult.

Figure 4 shows the next page of user input. After the users finish with setting up the constraints, the users will be shown the pairwise preferences, where they can choose the one they preferred than the other. In the figure, it shows the picture of outdoors vs amusement park. Without thinking any further, the users can just pick one from the two options available, or skip this pair if none of these matches their preferences. In this stage, the user can stop anytime and decide whether it is enough to show their preferences then proceed to see the result.

Figures 5 and 6 show the output of the recommender system. It shows the list of the most suitable POIs match with the user constraints and preference choices. If the result does not completely match the user preferences, they can save some of the spots and then keep shaking the rest choices. It also shows the summary of the trip destinations that also include the total expense, total entrance ticket price (*HTM/Harga Tiket Masuk*), total travel expense covering the cost of moving from one destination to another, and also total travel distance. It also shows the detail of each destination, the distance to the next destination and the estimated time spent in each of the particular destinations.

### Application layer

The backend (application) layer consists of three modules: (1) pairwise filtering, (2) genetic-based search, and (3) route optimizer. We will describe how each module works in more detail in this section.

The first module, *pairwise filtering*, works by limiting the available items to be selected as the first generation chromosome. We limit the items choice based on the place category is chosen by the user. For example, if the users select ‘outdoors’ and ‘beach’, then the system will limit the search only to all destinations under in these categories. In eliciting preferences, the interface can be turned into checkboxes as the most commonly applied in any online form. In this study, we introduce a pairwise approach, which is quite unusual for the users so we can observe their feedback.

The second part, *genetic-based search*, is the main module for this recommender system. In this study, we use a Genetic Algorithm (GA) as a search heuristic which is inspired by the theory of biological natural evolution. GA is used to find the best combination of destinations given the user constraints and preferences. The pseudocode of the search algorithm is shown in Algorithm 1.

Genetic Algorithm (GA) is a classical search algorithm that was developed based on the theory of biological evolution proposed by Charles Darwin by means of natural selection [27]. Darwin stated that the species with better fitness value will always win and survive in nature. These species of organisms evolve through the natural selection of inherited variations from their parents, which increases the individual’s ability to survive and compete. In the GA, the search is performed with a guidance of a fitness function and aims to produce a better generation (solution) through a series of the natural selection process, i.e. selection, cross-over, and mutation.

**Fig. 7:**
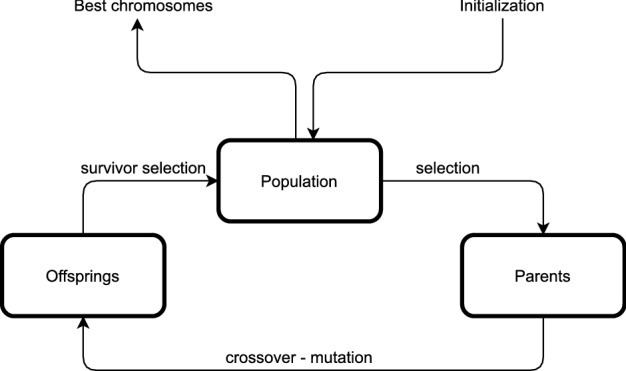
Genetic Algorithm Process

In [27] has been mentioned that the GA does not guarantee global maximum/minimum solution, but it often generates near-optimal solution instead. Therefore, the GA needs to be run multiple times until it achieves the desired results. The general process is illustrated in Fig. 7.

**Fig. 8:**
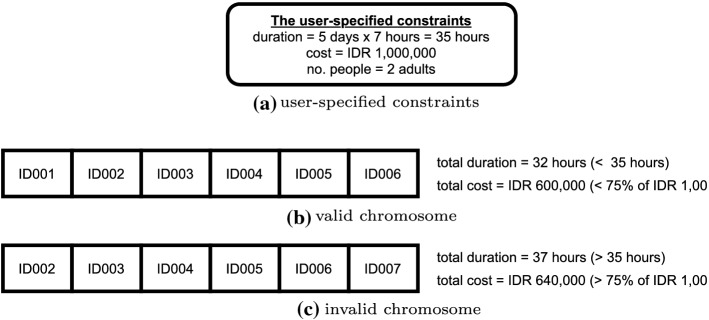
Chromosomes

The GA process consists of six main components as the following:

1. Chromosome and population generation
  Chromosomes consist of several genes. Chromosomes are used to represent a possible solution to a problem that will be solved using a genetic algorithm. The population in a genetic algorithm is a set of chromosomes. In a population, there will be *n* chromosomes with the value of *n* being a parameter set by the user. In our study, the gene is a single POI and a chromosome consists of many POIs. In the system, the gene data is represented with a POI’s ID.
  We only consider the chromosomes as valid if the total duration of exploring the place (how long a person stays in the place) and the total of entrance fees is still under the 75% cut-off point of the budget and 100% of the total duration (in hours), which has been specified by the users. In the end, we need to consider the travel cost as well, therefore we only use 75% of the budget as a valid requirement in this step. An example of what is considered as a valid chromosome is given in Figure 8, using the sample data given in Table 4.
2. Fitness function
  Every chromosome is assigned with a score calculated from a fitness function. The fitness function should reflect how close it is to the most optimal solution. In this study, we aim to achieve a closer match between the user-specified constraints and the total cost and duration find by the algorithm. We define our fitness function as below:
  1 $$\begin{aligned} \text {Fitness function} = \frac{1}{\text {cost score + duration score}} \end{aligned}$$
  where the ‘cost score’ is given by the difference between the user-specified budget and the total cost of the chromosomes, and the ‘duration score’ is given by the difference between the user-specified duration and the total duration of the chromosomes. The formula implies that the smaller the error/difference from the user-specified constraints, the higher the fitness value.
  The *cost score* calculation follows the formula below:
  2 $$\begin{aligned} \text {cost score} = \bigg |\frac{\text {budget} - (\sum _{i=1}^{n} \text {entrance fee}_i \times \text {no. of person}) + \text {travel exp.}}{100,000}\bigg | \end{aligned}$$
  where the ‘budget’ is the user-specified constraint of the budget, the ‘entrance fee’ is the cost of the entrance of each place obtain from the POIs database, *i* is the *i*-th place, *n* is the number of places, ‘no. of person’ is the user-specified constraint of the total adults joining in the group, and ‘travel exp.’ is the total travel cost. The formula takes the absolute error and normalizes it by using a factor of 100,000.
  For the total travel cost, we use a simple calculation assuming that for each kilometre, a taxi in Jakarta will cost IDR 5,000. We multiply the travel distances from one place to another, which can be obtained from the distance matrix database, with a cost of IDR 5,000. For example, from the distance matrix, we know that the distance between the place ID001 to the place ID002 is 5 km. So, the travel expense needed to move from place ID001 to place ID002 is 5km $\times$ IDR 5,000 = IDR 25,000.
  The *duration score* calculation is given by the formula given below:
  3 $$\begin{aligned} \text {duration score} =\left| \sum _{i=1}^{n} \text {stay duration}_i + \sum _{i=1}^{n-1} {\text {travel duration}}_i - \text {(duration} \times 10) \right| \end{aligned}$$
  where the ‘stay duration’ is the time spent by most people in each place obtained from the POIs database, ‘travel duration’ is the time of travelling from one place to another obtained from the distance matrix database, and ‘duration’ is the user-specified duration constraint in days. We multiply the duration by 10 because we assume the maximum time people can spend in a day for travelling is only 10 hours (9 am - 7 pm). So, again the formula takes the absolute error between the true duration and the outcome duration.
3. Parents selection The next important step in GA is to select two chromosomes as parents, which will be proceeded with the next step, i.e. crossover and mutation. In this study, we select the parents randomly.
4. Crossover and mutation
  Crossover is the process of crossing both chromosomes (as parents) to form a new offspring. Only valid offspring will be added to the existing population. The mutation process in GA is performed by replacing a gene with a new one. In our study, we only use a crossover process with a rate of 10 (every crossover is repeated 10 times), but zero rates for mutation. This number is based on several experiments conducted earlier, which were very obvious that the mutation process did not have any contribution to the fitness value improvement.
5. Survivor selection
  This process aims to select *n* chromosomes from the combination of the previous population with the resulting offsprings. Then, the selected *n* chromosomes will be used as a new generation of the population for the next iteration. We perform this step randomly and keep the size of the population to 40.
6. Stopping criteria
  The genetic algorithm will stop if there is a chromosome or a globally optimal solution found, the fitness value has reached convergence, or the iteration has reached the maximum number specified by the user. We set up the stopping criteria as below:
  Iteration rate = 8 (the whole cycle has been iterated for 8 times)
  Fitness score = 1.2 (this means the error rate is already very low < 1)

**Table 4:** Sample data

Place ID	Name	Entrance fee for	Total entrance cost	Stay Duration
		one adult (IDR)	for 2 adults (IDR)	(hours)
ID001	National Monument	5,000	10,000	2
ID002	Dufan Themepark	120,000	240,000	7
ID003	Museum Bank Indonesia	5,000	10,000	3
ID004	SS Waterpark TMII	20,000	40,000	6
ID005	Waterbom Pantai Indah Kapuk	130,000	260,000	7
ID006	Ragunan Zoo	20,000	40,000	7
ID007	Ants sculpture JGC	25,000	50,000	7

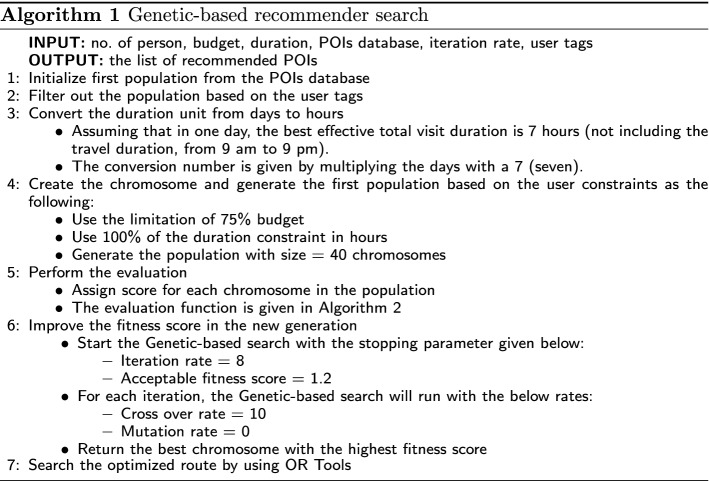

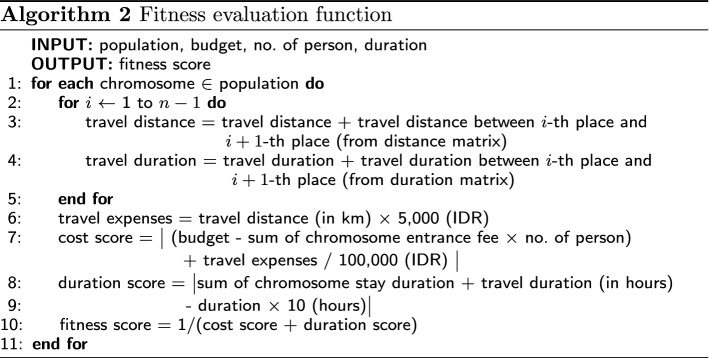





**Fig. 9:**
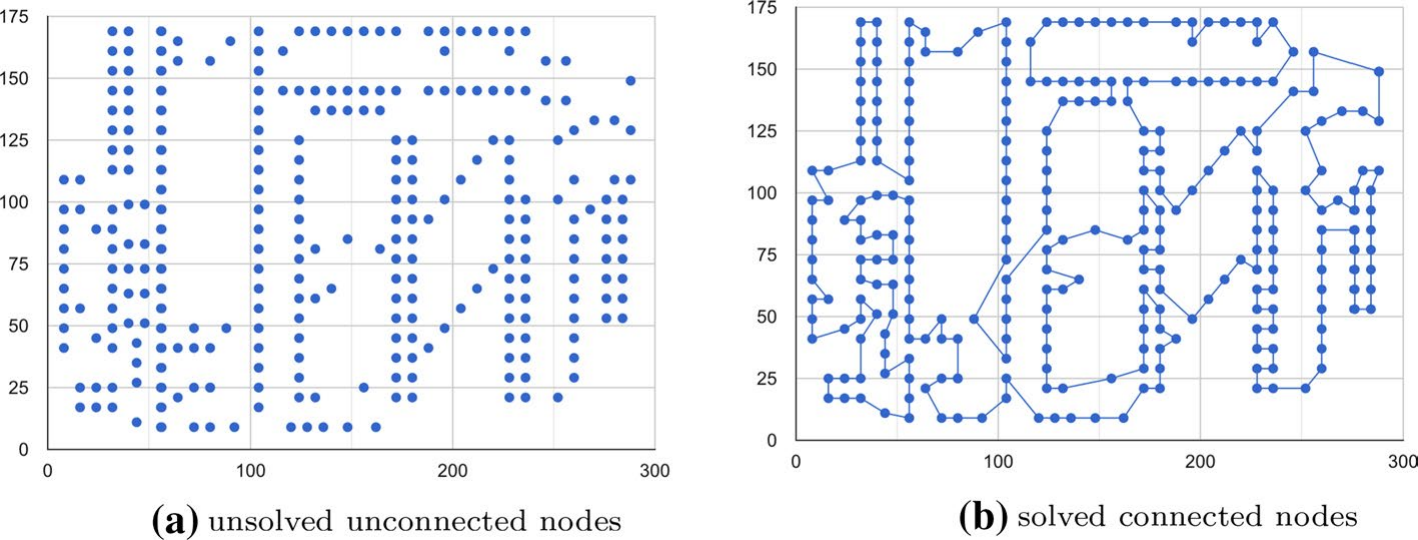
TSP problem solved with OR tools optimizer

**Fig. 10:**
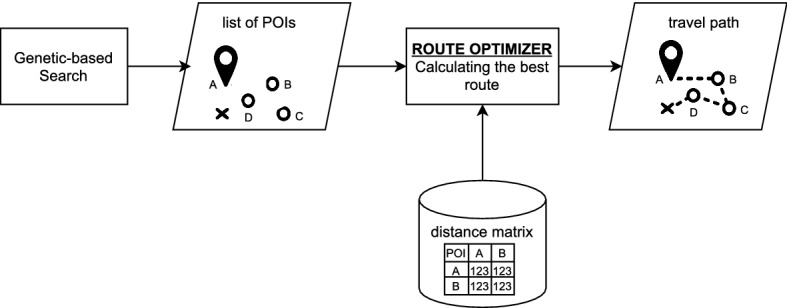
Route optimizer flow chart

The third module, *route optimizer*, is used to sort the destination based on the location and find the best-optimized route from start to finish. This module will work as the latest step in the recommender system. It takes the list of (unconnected) POIs from the GA-based search module as the input, and return the correct order of POIs. In this module, we implemented Google Optimization Tools (OR-Tools)^1^ which is an open source software for solving general combinatorial optimization problems. The OR-Tools library developer claims that they can solve the tour of 280 points in the plane very quickly, in less than a second on a typical computer. This case is shown in Figure 9. OR-Tools can be used for solving Travel Salesman Problem (TSP) in our case. The general TSP aims to find the shortest possible route to visit each city exactly once and return back to the origin city, when given a list of cities and the distances between each pair of cities. One needs the optimal route when planning the trip itinerary, otherwise it will result in time ineffectiveness. The system flow chart of the route optimizer is shown in Figure 10.

### Data layer

We store all the data in a NoSQL database so that the system can be easily scaled up for a larger dataset. The system use three databases as follows:

1. *Preference database*, to store the user choices.
  The user preferences are stored individually and will be considered for future search. The more data of users is collected the better the recommendation will be.
2. *POIs database*, to store the list of available POIs.
  In the current implementation, we only have POIs for one city which consists of 201 tourism POIs in Jakarta, Indonesia, but we will keep completing the database for future use.
3. *Distance matrix database*, to store the distance and the travel duration between each pair of the places.
  Google Maps use the unique name of the places and associate them with the geographical coordinate. By using the node names, we collected the optimal distance measure as well as the travel duration by using Google Maps API^2^. Google Maps calculate the distance by the most optimal route they found. The distance matrix data is used to calculate the total duration of the trip and optimizing it. It is also used by the route optimizer module in the system. The sample data is shown in Table 2 and 3.

## System evaluation

We implemented the trip recommender system and evaluate the system by using both offline and online experiment. For the offline experiment, we tried out the algorithm with default values as the inputs several times. The algorithm was executed on a machine with 8GB RAM with a 2.9 GHz Intel Core i5 processor. We show the report of the last 10 experiments average fitness value and average time execution in Table 5. The result shows that the algorithm average fitness is 0.2773 and the time execution is 0.0146 seconds. We also evaluate the result manually and all the list produced by the algorithm have good quality.

**Table 5:** Offline experiment result

	Fitness	Execution time (seconds)
	0.5207	0.0165
	0.1980	0.0008
	0.1508	0.0158
	0.3234	0.0165
	0.1893	0.0159
	0.2340	0.0158
	0.3881	0.0164
	0.2910	0.0162
	0.1668	0.0159
	0.3111	0.0157
Average	0.2773	0.0146
Minimum	0.1508	0.0008
Maximum	0.5207	0.0165
Std. deviation	0.1150	0.0048

We evaluated the system by using an online experiment. We used the standard evaluation framework for the recommender systems, known as ResQue (Recommender systems’ Quality of user experience) [28]. The framework aims to measure the recommended items quality, the system’s usability, usefulness, interface and interaction qualities, users’ satisfaction with the systems, and the influence of these qualities on users’ behavioral intentions. The questionnaire is shown in Figs. 13 and 14 in the Appendix. We also collected the demography of the participants, including age, gender, how often they go travelling, and how long they have been living in Jakarta. There are a total of 17 questions in the survey.

### Demography

**Fig. 11:**
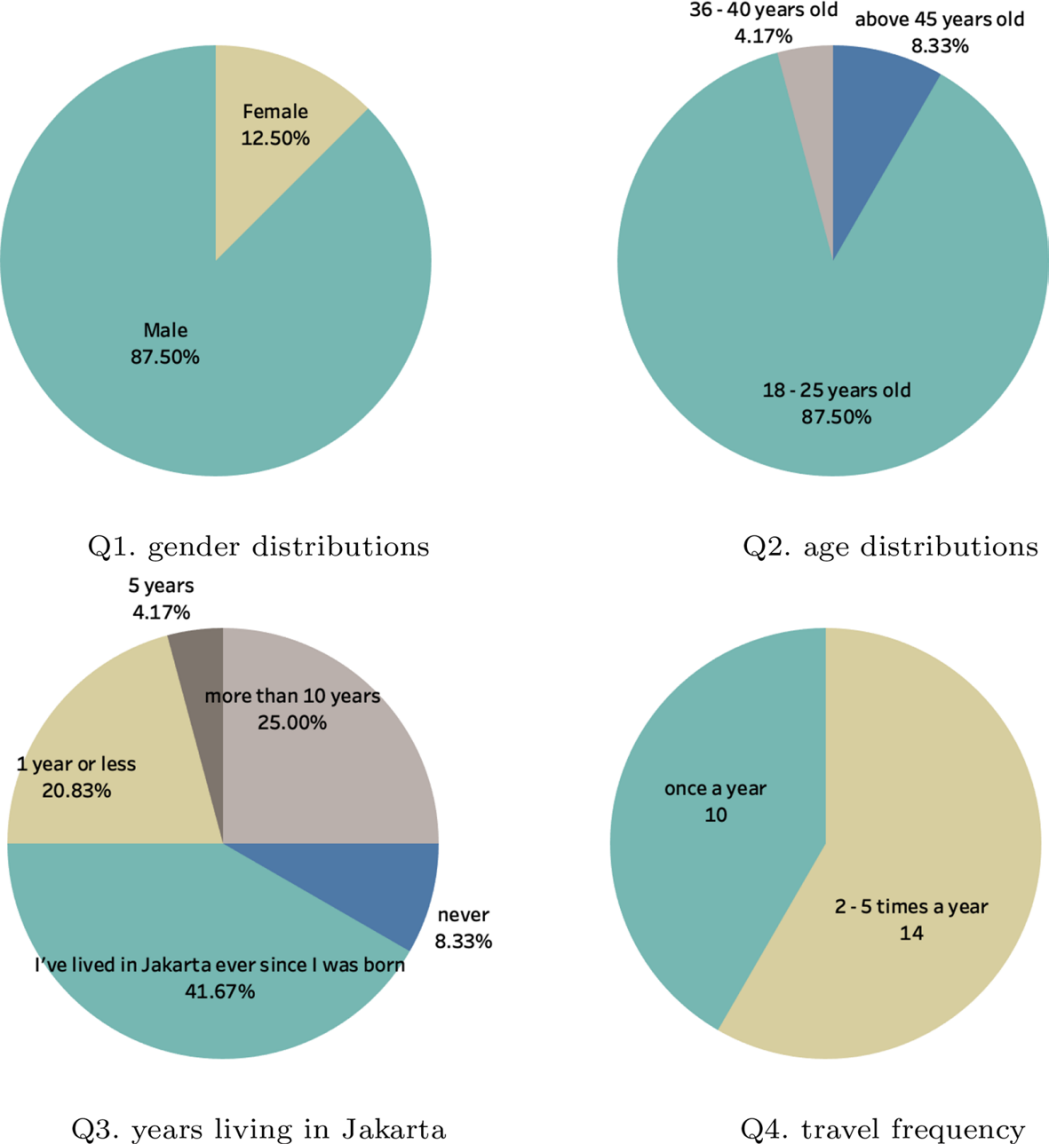
Demography Report

There were 24 participants recruited for this study. The questionnaire was distributed via e-mail and the guideline was also provided. The demography result can be seen in Figure 11. It is shown that from the gender, the male was the majority of the respondents when compared to the female proportions.

### Quality of the recommender system

**Fig. 12:**
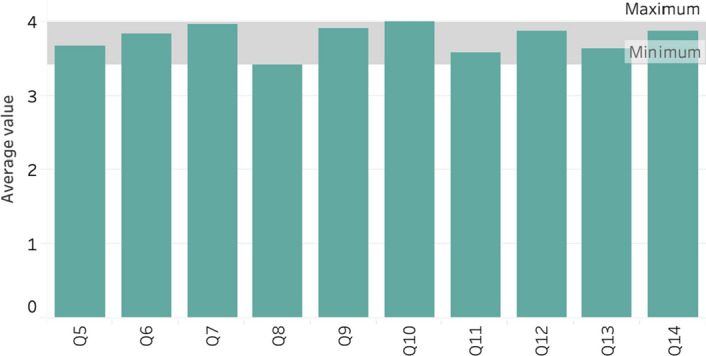
Average score of each question

**Fig. 13:**
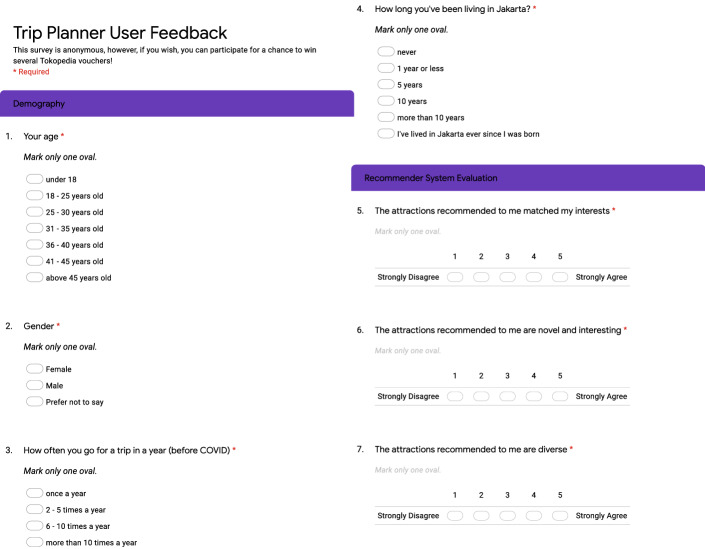
User evaluation questionnaire

**Fig. 14:**
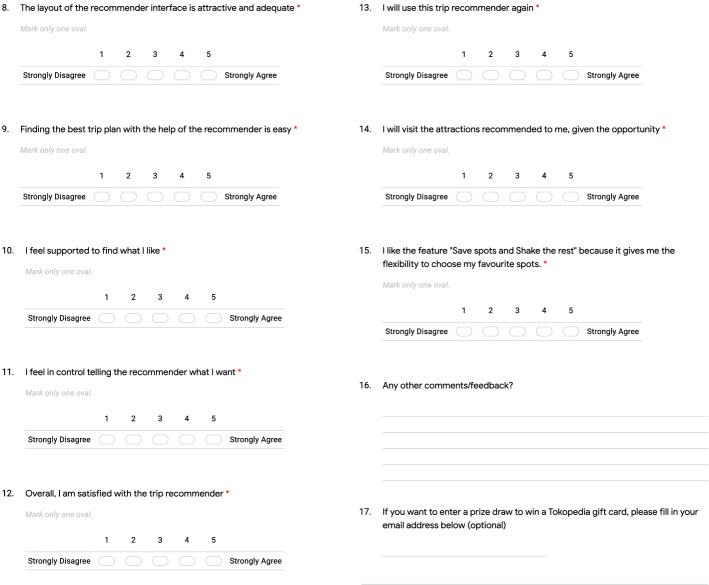
User evaluation questionnaire (cont.)

**Fig. 15:**
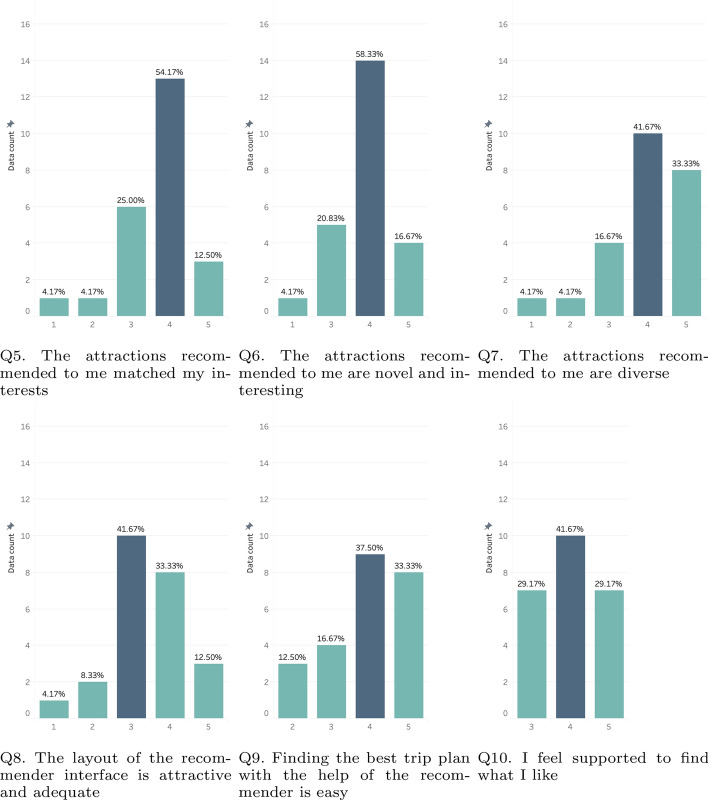
Questionnaire result

**Fig. 16:**
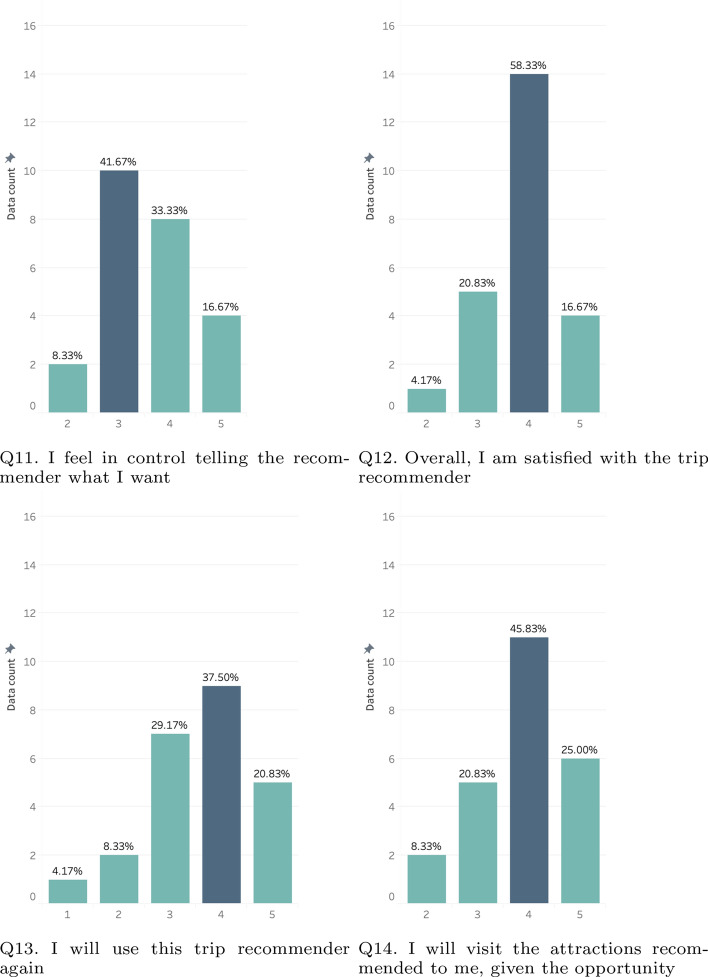
Questionnaire result (cont.)

This part consists of 10 questions, which measure the quality of the recommendation and user behaviour, following the ResQue framework mentioned earlier. The detailed result of each question is shown in Figure 15 and 16 in the Appendix. The average score of all the recommendation quality is shown in Figure 12, while the detail can be read from Table 6. From the result, we can observe the top three average were found in Q7 (3.96), Q9 (3.92) and Q10 (4.00). The Q7 was asking about the recommendation diversity, Q9 was about the easiness of finding the best trip plan and Q10 was questioning about the support given by the system to find what user wants. We can argue that these three aspects are actually the best features of the proposed system. On the other hand, the system seems very lack of good layout and design, as reflected in the Q8, which received the lowest average score (3.42) in the questionnaire.

**Table 6:** Questionnaire result

	Q5	Q6	Q7	Q8	Q9	Q10	Q11	Q12	Q13	Q14
Average	3.67	3.83	3.96	3.42	3.92	4.00	3.58	3.88	3.63	3.88
Std. deviation	0.92	0.87	1.04	0.97	1.02	0.78	0.88	0.74	1.06	0.90

### Additional feedback

The were last three questions (Q15, Q16 and Q17) were asked as additional feedback from the users. The first question was asking about the feature ‘save spot and shake the rest’ where the users can save some interesting spots and search the rest. We were also asking for any open feedback from the users in general about the system. We also provided an optional field for the users to put their email if they wish to be included in the raffle prize.

The answer result of the Q15. “I like the feature ‘Save spots and Shake the rest’ because it gives me the flexibility to choose my favourite spots.” gives an average of 4.29 with the standard deviation of 0.69. We can say that the users like the additional feature in the system.

The users also provided some additional feedback to the system by responding to the Q16 “Any other comments/feedback?”. We summarize the general feedbacks as listed below:

- There are still few mountain destinations
- The only major downside of the system is that the category selection is limited
- It is difficult to understand that the users can stop anytime and get the results straight away
- The interface can be more descriptive
- Some features need more clarity
- The system still lacks textual explanation to guide the users
- Some of the image resolution were out of aspect ratio.
- The application is really easy to use

Based on some feedback given by the users which were mostly about the layout and textual clarity, we need to work more on the design for future development.

## Conclusion and future work

In this paper, we have shown the design implementation of a trip planner recommender system that employs the genetic algorithm as the search method combined with pairwise elicitation preferences. We have proposed the approach which has successfully implemented as a working web-based application with three-layer architecture. We have also published an attraction dataset that can be used for further study. The evaluation of the system shows a very satisfying result, although some improvement may still need to be performed, such as designing an adequate and interactive layout. Feedback from the user was also collected in the form of a survey which can be used for future development. We plan to improve the algorithm by weighting the places with a higher rating more than the ones with a lower rating. We also need to work on the interface design to make sure the user understand how to use the pairwise feature. This approach can also be expanded for other cities with a more complete place labelling and more accurate data of travel cost. When a more complete dataset has been obtained, then the performance of the proposed approach can be evaluated on a bigger dataset.

## Data Availability

The implementation of the proposed algorithm in Python 3 and the dataset used are available at https://github.com/nnqomariyah under GNU General Public License v3.0. Please cite this paper to reuse the dataset and/or modify the code.

## Appendix

See Figs. 13, 14, 15, and 16.

## Footnotes

1 https://github.com/google/or-tools.

2 https://developers.google.com/maps/documentation/distance-matrix.
